# Antibiotic Choices for Pediatric Periorbital Cellulitis—A 20-Year Retrospective Study from Taiwan

**DOI:** 10.3390/antibiotics11101288

**Published:** 2022-09-21

**Authors:** En-Jie Shih, Jui-Kuang Chen, Pei-Jhen Tsai, Muh-Chiou Lin, Youn-Shen Bee

**Affiliations:** 1 Department of Ophthalmology, Kaohsiung Veterans General Hospital, Kaohsiung 81362, Taiwan; 2 School of Medicine, National Yang Ming Chiao Tung University, Hsinchu 30068, Taiwan; 3 Division of Infectious Diseases, Kaohsiung Veterans General Hospital, Kaohsiung 81362, Taiwan; 4 Institute of Biomedical Sciences, National Sun Yat-sen University, Kaohsiung 80424, Taiwan; 5 Department of Optometry, Shu-Zen Junior College of Medicine and Management, Kaohsiung 82144, Taiwan

**Keywords:** pediatric periorbital cellulitis, etiologies, bacteria, drug susceptibility tests, antibiotics

## Abstract

The delayed treatment of pediatric periorbital cellulitis may have severe consequences. In addition, the antibiotic efficacy against causative bacteria may change over time, and it is important to understand the appropriate antibiotic options for effective treatment in pediatric patients. We compared the changes in cultured bacteria and drug susceptibility tests between two decades, 2010–2019 and 2000–2009, to establish antibiotics for empirical use. The patient characteristics, etiologies, culture sites, and isolated bacteria, and the antibiotic susceptibility tests of the admitted pediatric patients (*n* = 207) diagnosed with preseptal and orbital cellulitis during 2000 to 2019, were recorded. Insect/animal bites (*p* = 0.084) showed an increasing trend, and sinusitis (*p* = 0.016) showed a significant decrease in the past decades. The most common bacteria were *Staphylococcus aureus*, and methicillin-resistant *S. aureus* (MRSA) infections increased in recent decades (*p* = 0.01). Moreover, we found that vancomycin was ideal for MRSA infections. The decreasing efficacy of oxacillin correlates with the increasing proportion of MRSA in pediatric periorbital cellulitis. Our study thus offers antibiotic choices against the most common isolates that can be administered before culture results are available.

## 1. Introduction

Periorbital cellulitis is categorized as either preseptal or orbital cellulitis, which are defined as infections of the subcutaneous tissue anterior to the orbital septum and soft tissue posterior to the septum, respectively [1,2,3]. Recent studies on periorbital cellulitis in children have focused mainly on patient characteristics, clinical presentations, laboratory findings, and treatment effectiveness [1,2,3,4,5,6]. For example, a higher incidence of orbital cellulitis in males over females has been reported [7], and pediatric preseptal cellulitis is more common in younger patients than orbital cellulitis [1,2,4]. Moreover, seasonal distribution of pediatric periorbital cellulitis is reportedly associated with specific etiologies, such as upper respiratory infection and sinusitis [2,8,9]. Pediatric orbital cellulitis is significantly more common in the winter, while pediatric preseptal cellulitis is significantly more common in the spring [2,4,8].

Apart from seasonal variations in the incidence of periorbital cellulitis, previous studies have also demonstrated the relationship between predisposing factors and bacterial infection. Sinusitis is the most common source of periorbital cellulitis, especially in orbital infections, with *Streptococcus pneumoniae* and *Streptococcus anginosus* as the primary causes [2,4,10]. The normal flora of the skin, which includes *Staphylococcus aureus* (*S. aureus*) and *Streptococcus pyogenes*, are important etiologic agents in preseptal cellulitis, and direct inoculation can occur through an insect/animal bite, skin infection, or trauma [4,5]. Due to the introduction of the *Haemophilus influenzae* type b (Hib) vaccine in 1985, the incidence of Hib orbital cellulitis has dramatically decreased along with the related severe complications, such as bacteremia, sepsis, and meningitis [3,5,10].

Although the mortality rate of pediatric orbital cellulitis reduced from 17% to 2.5% after the introduction of antibiotics, blindness still occurs in 11% of children [3]. Previous studies have recommended the administration of empirical antibiotics that fight common pathogens to patients with periorbital cellulitis before culture results are available [3,5,10,11,12]. However, as the universal use of antibiotics has increased, a rise in antibiotic resistance of isolates in periorbital cellulitis has been noted [10,13]. Thus, the analysis of antibiotic efficacy against pediatric periorbital cellulitis in recent years is necessary for better prognosis and avoidance of severe complications. To the best of our knowledge, few studies have compared the changes in cultured bacteria and drug susceptibility tests in pediatric periorbital cellulitis over time. We hypothesized that the composition of causative bacteria and antibiotic effectiveness in periorbital cellulitis has changed over the past two decades. Physicians may adjust antibiotic use according to antimicrobial susceptibilities. We collected data from children with preseptal or orbital cellulitis admitted to our hospital during 2000–2009 and 2010–2019 and analyzed the differences in patient characteristics, etiologies, cultured bacteria, and drug susceptibility tests against common isolates in the two decades.

## 2. Results

### 2.1. Demographics and Characteristics of Patients

A total of 207 pediatric patients were recruited (2000–2009, *n* = 105; 2010–2019, *n* = 102). From January 2000 to December 2009, there were 92 and 13 cases of preseptal and orbital cellulitis, respectively, and from January 2010 to December 2019, there were 88 and 14 patients with preseptal and orbital cellulitis, respectively. Patient characteristics and etiologies are presented in Table 1.

**Figure 1:**
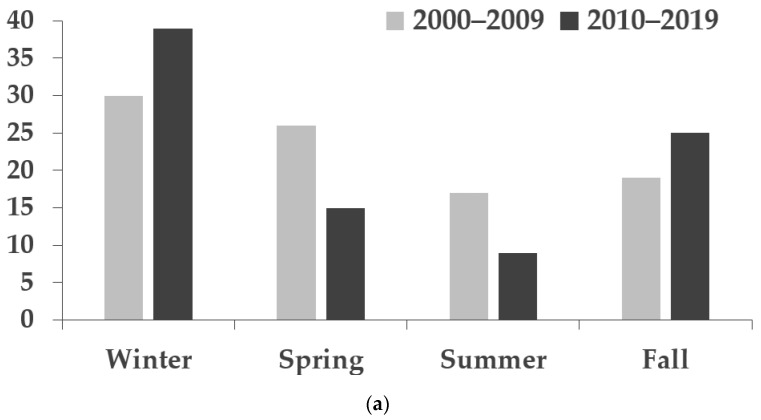

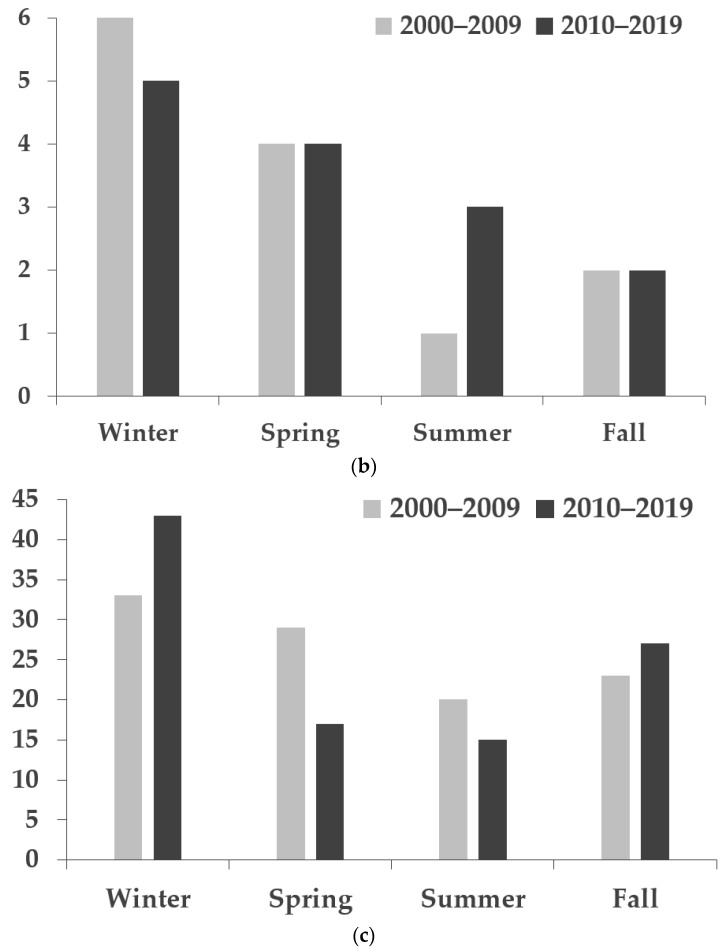
Seasonal distribution of patients with (**a**) preseptal cellulitis, (**b**) orbital cellulitis, and (**c**) preseptal plus orbital cellulitis.

A higher percentage of males than females were diagnosed with orbital cellulitis in both 2000–2009 (53.9%) and 2010–2019 (64.3%). The average ages of patients with orbital cellulitis were higher than those of patients with preseptal cellulitis in both decades (preseptal cellulitis: 3.5 ± 3.8 vs. 4.1 ± 4.3; orbital cellulitis: 5.0 ± 4.0 vs. 6.3 ± 5.6 in 2000–2009 and 2010–2019, respectively). For the overall population, the average ages were 3.7 (± 3.8) and 4.4 (± 4.5) years, and the median ages were 2 (1–6) and 2.5 (1.4–6) years in the two decades, respectively. The seasonal distribution of periorbital cellulitis showed an increased incidence during the winter months of December, January, and February for both preseptal and orbital cellulitis (Figure 1). There were no significant differences according to sex (*p* = 0.141), eye (*p* = 0.942), age (*p* = 0.212), and seasonal distribution (*p* = 0.146) between 2000–2009 and 2010–2019.

The causes of infection that could not be traced back from the medical records were identified as unknown causes. The most common etiologies were hordeolum, insect/animal bite, and upper respiratory infection. The proportion of insect/animal bites showed an increasing trend from 2000–2009 to 2010–2019; conversely, the percentage of patients with sinusitis decreased significantly from 2000–2009 to 2010–2019.

### 2.2. The Culture Results from Different Culture Sites

The culture sites and positive culture rates are listed in Table 2. The positive culture rate in local culture from abscesses via incision and drainage (75.0–100%) was higher than that of culture from conjunctival swabs (60.0–66.7%) in both decades. Systemic culture from blood samples was requested in 61.5–92.9% of patients in different subgroups and was sterile in most cases (97.1–100%). In total, 2 of 70 (during 2000–2009) and 1 of 78 (during 2010–2019) blood cultures showed positive results for preseptal cellulitis. The isolated pathogens in the blood samples were coagulase-negative staphylococci (CoNS) and *Staphylococcus hominis.*

The numerator represents the number of positive cultures, and the denominator represents the total number of cultures performed at each site.

### 2.3. The Cultured Bacteria in Preseptal and Orbital Cellulitis

The most common isolated pathogens were methicillin-susceptible *Staphylococcus aureus* (MSSA) and methicillin-resistant *Staphylococcus aureus* (MRSA), which accounted for 61.2% and 72.0% of bacterial infections in 2000–2009 and 2010–2019, respectively (Table 3). In addition, the percentage of MSSA showed a decreasing trend (*p* = 0.055) over time, and the proportion of MRSA increased significantly (*p* = 0.01) over time. Other staphylococcal species (CoNS, *Staphylococcus epidermidis* and *S. hominis)* and streptococcal species (*Streptococcus constellatus, Streptococcus viridians, Streptococcus pneumoniae,* and group A *Streptococcus*) were recorded.

Our study revealed 2 of 105 (during 2000–2009) and 1 of 102 (during 2010–2019) cases with polymicrobial culture results. The cultured bacteria included MSSA, CoNS, *S. constellatus, S. viridans, H. influenzae, Citrobacter koseri,* and *Eikenella corrodens.* In total, there were 16 organisms isolated in our study, which are listed in Table 4.

Susceptibility testing against MSSA and MRSA (Table 5) showed that imipenem, levofloxacin, rifampin, vancomycin, and trimethoprim/sulfamethoxazole had a sensitivity >80% against MSSA and MRSA during both 2000–2009 and 2010–2019. Conversely, oxacillin showed reduced efficacy against S. aureus from 2000–2009 to 2010–2019.

## 3. Discussion

In children with red and painful eyes, it is important to differentiate periorbital cellulitis from other orbital inflammatory diseases such as contact dermatitis, allergic conjunctivitis, and idiopathic orbital inflammatory disease [3]. Timely and effective antibiotic treatment is necessary to avoid life-threatening complications and profound visual loss in pediatric periorbital cellulitis, especially in orbital cellulitis [4,5,14]. Previous pediatric studies have demonstrated a male predominance in both preseptal and orbital cellulitis, with the percentage of males varying from 56% to 90% [4,15,16,17,18]. In our study, a higher proportion of males (52.2–64.3%) was noted in the preseptal cellulitis group during the 2000–2009 period and in the orbital cellulitis group in both time periods. Previous studies have also reported that the mean age of pediatric preseptal cellulitis patients is lower (3.5–5.4 years) than that of orbital cellulitis patients (5.5–9.4 years) [1,4,11], and the median age of children with periorbital cellulitis is 5–6 years [1,2,19]. Our study showed similar results: the average age of preseptal cellulitis patients was lower than that of orbital cellulitis patients during both the 2000–2009 and 2010–2019 periods.

The seasonal distribution of pediatric periorbital cellulitis shows a peak in winter, which coincides with the peak time for development of common etiologies, such as sinusitis and upper respiratory infection [2,4,15,20]. These findings were confirmed in our study in that a peak was noted in the winter for both types of cellulitis.

Most previous studies report sinusitis as the most common pathophysiology of pediatric periorbital cellulitis (28.6–90%), especially post-septal cellulitis [2,3,4,5,6,10,11,21,22]. Other causes of preseptal cellulitis include insect bites, head or facial injuries, and dental abscesses. The causes of orbital cellulitis include external ocular infections such as dacryocystitis, dacryoadenitis, hordeolum, and conjunctivitis [2,4,23,24]. In our study, sinusitis was also a common cause of periorbital infection in the 2000–2009 peroid, but it was a significantly less common cause in the 2010–2019 period (*p* = 0.016). A decreasing proportion of sinusitis was also found in our recent study of periorbital infections in adults during the 2000–2019 period [25]. A further evaluation of whether the incidence of sinusitis is actually decreasing or the treatment of the disease is more effective in Taiwan, thus avoiding the development of periorbital cellulitis, is necessary. In this pediatric study, hordeolum, insect/animal bite, and upper respiratory infection were the most common predisposing factors, accounting for nearly 50% of periorbital infections in both decades.

In this study, local culture obtained via incision and drainage of abscesses showed a higher positive culture rate than that obtained via conjunctival swabs. Blood culture results were sterile in most of the samples. This finding was consistent with previous periorbital studies showing higher positive culture rates in surgical wounds and lower positive culture rates from eye discharge or blood collection [2,3,4,7,10,18,19,26]. Children hospitalized with periorbital cellulitis are usually treated with aggressive intravenous antibiotic therapy and undergo evaluation for identification of bacteremia in blood cultures [27,28]. However, given the extremely low positive blood culture rate demonstrated in our study (0–2.9%), routine blood collection could be avoided in periorbital cellulitis.

Previous studies have reported staphylococcal species, streptococcal species, and *H. influenzae* as common bacteria involved in pediatric periorbital cellulitis [2,8,29]. Among these species, *S. aureus* is the most common isolate but whether the proportion of MRSA infections increases or not has been controversial [19,26]. *S. aureus, S. pneumonia*, *S. pyogenes,* and *H. influenzae* tend to reflect the association between periorbital cellulitis and sinusitis or traumatic causes [23,24]. *S. anginosus* could be pathological as part of the upper respiratory flora [19]. Due to the universal vaccinations against *H. influenzae*, an apparent reduction in Hib orbital cellulitis was observed.

Our study showed that the most common pathogen observed was *S. aureus*, followed by other staphylococcal and streptococcal species. The ratio of MRSA strains to all *S. aureus* strains increased from 50% during the 2000–2009 period to 88.9% during the 2010–2019 period. This trend was consistent with previous studies in Taiwan which indicated that the rate of MRSA infection increased from 20.2% in the 1981–1986 period to 69.3% in 1999 [30,31]. In our recent study of periorbital infections in adults, the proportion of MRSA strains also increased from 39.5% during the 2000–2009 period to 65.6% during the 2010–2019 period [25]. According to a previous study, the most frequently cultured microbes from sinusitis in Taiwan and other countries were *S. aureus*, CoNS, and streptococcal species such as *S. pneumoniae* and *S. viridans* [32,33,34]. Thus, in our study, fewer streptococcal species might correlate with significantly decreasing cases of sinusitis from 2000–2009 to 2010–2019.

Our culture results are consistent with those of previous studies showing that periorbital cellulitis in children tends to show a single bacterial infection compared with polymicrobial results [4,5]. Since the most common organisms are streptococcal and staphylococcal species, empirical antibiotics should target these pathogens [10]. In MRSA cases, vancomycin should be considered [10,35]. On the other hand, if the culture results show MSSA infection, vancomycin should be changed to beta-lactam combined with a beta-lactamase inhibitor such as amoxicillin/clavulanic acid or first-generation cefalosporins [36,37,38]. The reducing effectiveness of oxacillin in our drug susceptibility testing is consistent with the increase in MRSA infections in recent decades. Sensitivity testing for other frequently used antibiotics against MSSA, including amoxicillin/clavulanic acid and ampicillin/sulbactam, with sensitivity rates of 54.6% and 33.3%, respectively, were performed during the 2000–2009 period but not during the 2010–2019 period. Antimicrobial susceptibility testing for other antibiotics recommended for periorbital cellulitis, such as first- and third-generation cephalosporins (cefazolin, cefotaxime, and ceftazidime) with a sensitivity rate ranging from 55.6% to 100%, was also performed in the 2000–2009 period but ceased in the 2010–2019 period. This is due to the policy of our microbiological laboratory that sensitivity tests for amoxicillin/clavulanic acid, ampicillin/sulbactam, and cephalosporines, which are not usually used for MRSA infection, are not performed. In our study, rifampin showed a high sensitivity rate of 100% in both patient groups; however, since Taiwan has a high tuberculosis incidence rate, and a study in 2011 showed that the incidence of tuberculosis in patients <20 years of age was 9.61/100,000 person-years, rifampin is usually avoided in other non-tuberculosis infections to prevent delayed diagnosis and drug resistance induced by partial treatment of tuberculosis [39]. Based on the high sensitivity rate found in our study, oral forms of trimethoprim/sulfamethoxazole and minocycline could be considered for patients with improved symptoms and those ready to be discharged. Although it has a good sensitivity rate, levofloxacin, as fluoroquinolone antibiotics, is usually not recommended for use in children due to musculoskeletal adverse effects [40]. If the culture results show mixed flora and not just Gram-positive bacteria, broad-spectrum intravenous antibiotics are still needed to prevent infections caused by other Gram-negative bacteria and anaerobes before the culture results are available [5]. In children, beta-lactam combined with a beta-lactamase inhibitor such as amoxicillin/clavulanic acid or piperacillin/tazobactam could be chosen to treat both aerobic and anaerobic infections [38]. In preseptal cellulitis, ciprofloxacin and clindamycin can be used for children allergic to penicillin; furthermore, triple antibiotic treatment comprising cefotaxime, flucloxacillin, and metronidazole can be applied in orbital cellulitis [3]. Additionally, clindamycin and metronidazole can cover anaerobes [41,42,43]. Apart from cefotaxime, cephalosporins such as ceftriaxone and ceftazidime, which have adequate cerebrospinal fluid penetration, are other options for orbital cellulitis [10,19,42].

The limitations of our study are that we excluded patients with preseptal cellulitis with a satisfactory response to oral antibiotics at the outpatient department rather than admitting them for intravenous antibiotics. In addition, the findings of sensitivity analyses in Taiwan may not be applicable to other regions because the prevalence of bacterial infection and the use/abuse of antibiotics can vary remarkably. Based on the above considerations, our study has a selection bias.

The strength of this study is that we analyzed the change in bacterial infections and the effectiveness of antibiotics in pediatric periorbital cellulitis during the 2000–2009 period and the 2010–2019 period. To the best of our knowledge, few studies discussing periorbital infection in children have compared the differences in isolates and antimicrobial susceptibility testing over successive decades. In addition, the analysis of patient characteristics and etiologies provides some practical information for physicians to evaluate children with red and swollen eyes.

## 4. Materials and Methods

### 4.1. Participants and Ethics

This retrospective, cross-sectional study recruited patients younger than 18 years of age admitted to Kaohsiung Veterans General Hospital (KSVGH) between January 2000 and December 2019 due to preseptal or orbital cellulitis. The patients were identified using the International Classification of Diseases, Ninth Revision (ICD-9) codes for abscess of the eyelid (373.13), orbital cellulitis (376.01), cellulitis and abscess of the face (682.0), and those with ICD-10 codes for cellulitis of the right orbit (H05.011), cellulitis of the left orbit (H05.012), cellulitis of the bilateral orbits (H05.013), cellulitis of unspecific orbits (H05.019), and periorbital cellulitis (L03.213). Our study was performed according to the tenets of the Declaration of Helsinki, and the study protocol was approved by the Institutional Review Board (KSVGH20-CT4-05) of KSVGH in Taiwan. Due to its retrospective nature, our study meets the criteria for a waiver of informed consent in accordance with the institutional requirements: the waiver will not adversely affect the rights of the patients, and the research involves no more than minimal risk to them.

### 4.2. Data Retrieval and Processing

Periorbital patients were classified according to diagnosis of preseptal or orbital cellulitis and time of admission during the 2000–2009 and 2010–2019 periods. The hospitalized patients received empirical antibiotics first and the antibiotics might be changed according to clinical response, culture results, or reports of drug susceptibility tests. If the disease progressed even under antibiotic therapy according to the physical examinations, laboratory data, or image findings, further surgical interventions such as incision and drainage of abscesses, functional endoscopic sinus surgery for sinusitis, and extraction of caries were performed.

We recorded and analyzed data on sex, eyes, age, months, and seasons at the time of admission. The seasons were defined as spring (March, April, and May), summer (June, July, and August), fall (September, October, and November), and winter (December, January, and February). The etiologies of periorbital cellulitis were assessed according to history, clinical examination, and images from the medical records.

The sites of sample collection, isolated pathogens, and results of antibiotic susceptibility testing for the most common isolates were evaluated. The locations of sample collection were classified as local (conjunctival swab, abscess via incision and drainage, and nasopharyngeal swab), or systemic (blood) culture. Because our goal was to assess the efficacy of cultured bacteria and antibiotics against pediatric periorbital infection, we excluded patients with viral or fungal infections. Our laboratory does not routinely conduct drug susceptibility tests for anaerobes or bacterial species that have not been recommended in the guidelines of the Clinical & Laboratory Standards Institute (CLSI).

The selection of antibiotics for susceptibility testing was based on the guidelines of the CLSI, the available antibiotics from the policy of the Taiwan Centers for Disease Control, and the consensus according to the Infection Control and Pharmacy Department in KSVGH. The performance and interpretation of antimicrobial susceptibility testing were based on CLSI recommendations.

### 4.3. Statistical Analyses

The data were analyzed using SPSS 22.0 (IBM Corp., Armonk, NY, USA). The sum, percentage, average, standard deviation, and median of the variables were calculated. The chi-squared test/Fisher’s exact test, and Student’s t-test were used to analyze categorical and continuous characteristics, respectively. Statistical significance was set at *p* < 0.05.

## 5. Conclusions

The most commonly cultured bacteria in pediatric periorbital cellilutis were *S. aureus.* An increase in MRSA infections is consistent with reduced oxacillin efficacy against periorbital cellulitis in recent decades. The drug sensitivity test findings offer antibiotic choices for preseptal and orbital cellulitis in children that can be administered before culture results are available.

## Figures and Tables

**Table 1:** Demographics and characteristics of patients with preseptal or orbital cellulitis.

	2000–2009			2010–2019			
	Preseptal Cellulitis (*n* = 92)	Orbital Cellulitis (*n* = 13)	Total (*n* = 105)	Preseptal Cellulitis (*n* = 88)	Orbital Cellulitis (*n* = 14)	Total (*n* = 102)	*p*-Value
**Sex (%) ^a^**							
**Male**	48 (52.2)	7 (53.9)	55 (52.4)	34 (38.6)	9 (64.3)	43 (42.2)	0.141
**Female**	44 (47.8)	6 (46.2)	50 (47.6)	54 (61.4)	5 (35.7)	59 (57.8)	
**Eye (%) ^a^**							
**Right**	37 (40.2)	3 (23.1)	40 (38.1)	30 (34.1)	8 (57.1)	38 (37.3)	0.942
**Left**	50 (54.3)	9 (69.2)	59 (56.2)	52 (59.1)	5 (35.7)	57 (55.9)	
**Both**	5 (5.4)	1 (7.7)	6 (5.7)	6 (6.8)	1 (7.1)	7 (6.9)	
**Age (%) ^b^**							
**Mean ± SD**	3.5 ± 3.8	5.0 ± 4.0	3.7 ± 3.8	4.1 ± 4.3	6.3 ± 5.6	4.4 ± 4.5	0.212
**Median (IQR)**	2 (1.0–4.8)	6 (0.5–8.5)	2 (1.0–6.0)	2.3 (1.3–4.8)	5 (1.5–10.3)	2.5 (1.4–6.0)	
**Etiology (%) ^a^**							
**Dacryocystitis**	2 (2.2)	0 (0.0)	2 (1.9)	1 (1.1)	0 (0.0)	1 (1.0)	1.000
**Dacryoadenitis**	0 (0.0)	0 (0.0)	0 (0.0)	0 (0.0)	1 (7.1)	1 (1.0)	0.493
**Hordeolum**	16 (17.4)	1 (7.7)	17 (16.2)	13 (14.8)	1 (7.1)	14 (13.7)	0.619
**Conjunctivitis**	3 (3.3)	0 (0.0)	3 (2.9)	5 (5.7)	2 (14.3)	7 (6.9)	0.210
**Insect/animal bite**	16 (17.4)	0 (0.0)	16 (15.2)	17 (19.3)	6 (42.9)	23 (22.5)	0.084
**URI**	17 (18.5)	0 (0.0)	17 (16.2)	16 (18.2)	2 (14.3)	18 (17.6)	0.780
**Skin infection**	9 (9.8)	0 (0.0)	9 (8.6)	8 (9.1)	1 (7.1)	9 (8.8)	0.849
**Sinusitis**	10 (10.9)	4 (30.8)	14 (13.3)	5 (5.7)	0 (0.0)	5 (4.9)	0.016
**Odontogenic origin**	2 (2.2)	2 (15.4)	4 (3.8)	4 (4.5)	0 (0.0)	4 (3.9)	1.000
**Periocular trauma**	3 (3.3)	0 (0.0)	3 (2.9)	2 (2.3)	0 (0.0)	2 (2.0)	1.000
**Acute tonsillitis**	1 (1.1)	0 (0.0)	1 (1.0)	0 (0.0)	0 (0.0)	0 (0.0)	1.000
**Dermoid cyst rupture**	0 (0.0)	1 (7.7)	1 (1.0)	0 (0.0)	0 (0.0)	0 (0.0)	1.000
**Scarlet fever**	0 (0.0)	1 (7.7)	1 (1.0)	0 (0.0)	0 (0.0)	0 (0.0)	1.000
**Malignancy**	0 (0.0)	1 (7.7)	1 (1.0)	0 (0.0)	0 (0.0)	0 (0.0)	1.000
**Unknown causes**	13 (14.1)	3 (23.1)	16 (15.2)	17 (19.3)	1 (7.1)	18 (17.6)	0.640

^a^ The *p*-value was estimated using the chi-squared test or Fisher’s exact test, depending on the sample size. ^b^ The *p*-value was estimated using the Student’s *t*-test. Abbreviations: Ave, average; SD, standard deviation; IQR, interquartile range; URI, upper respiratory infection.

**Table 2:** Culture sites.

	2000–2009		2010–2019	
	Preseptal Cellulitis	Orbital Cellulitis	Preseptal Cellulitis	Orbital Cellulitis
**Local culture (%)**				
**Conjunctival swab**	9/14 (64.3)	3/5 (60.0)	8/12 (66.7)	2/3 (66.7)
**Abscess**	16/18 (88.9)	0 (0.0)	9/12 (75.0)	4/4 (100.0)
**Nasopharyngeal swab**	2/2 (100.0)	1/1 (100.0)	0 (0.0)	0 (0.0)
**Total**	27/34 (79.4)	4/6 (66.7)	17/24 (70.8)	6/7 (85.7)
**Systemic culture (%)**				
**Blood**	2/70 (2.9)	0/8 (0.0)	1/78 (1.3)	0/13 (0.0)

**Table 3:** Most common pathogens isolated: MSSA, MRSA, and other staphylococcal and streptococcal species.

	2000–2009			2010–2019			
	Preseptal Cellulitis (*n* = 31)	Orbital Cellulitis (*n* = 5)	Total (*n* = 36)	Preseptal Cellulitis (*n* = 19)	Orbital Cellulitis (*n* = 6)	Total (*n* = 25)	*p*-Value ^a^
Pathogen (%)							
MSSA	11 (35.5)	0	11 (30.6)	1 (5.3)	1 (16.7)	2 (8.0)	0.055
MRSA	10 (32.3)	1 (20.0)	11 (30.6)	11 (57.9)	5 (83.3)	16 (64.0)	0.010
Other staphylococcus species ^b^	5 (16.1)	0	5 (13.9)	3 (15.8)	0	3 (12.0)	1.000
Streptococcus species ^c^	2 (6.5)	2 (40.0)	4 (11.1)	0	0	0	0.137
Others	3 (9.7)	2 (40.0)	5 (13.9)	4 (21.1)	0	4 (16.0)	0.727

^a^ The *p*-value was estimated using the chi-squared test or Fisher’s exact test, depending on the sample size. ^b^ Other staphylococcal species include coagulase-negative staphylococci, *S. epidermidis* and *S. hominis.* ^c^ The streptococcal species include *S. constellatus, S. viridians, S. pneumonia,* and *group A Streptococcus*. Abbreviations: MSSA, methicillin-susceptible *Staphylococcus aureus*; MRSA, methicillin-resistant *Staphylococcus aureus.*

**Table 4:** All the pathogens isolated in periorbital cellulitis.

	2000–2009			2010–2019		
	Pre-Septal (%)	Orbital (%)	Total (%)	Pre-Septal (%)	Orbital (%)	Total (%)
	(*n* = 31)	(*n* = 5)	(*n* = 36)	(*n* = 19)	(*n* = 6)	(*n* = 25)
Pathogen (%)						
Gram positive						
MSSA	11 * (35.5)		11 * (30.6)	1 (5.3)	1 (16.7)	2 (8.0)
MRSA	10 (32.3)	1 (20.0)	11 (30.6)	11 (57.9)	5 (83.3)	16 (64.0)
CoNS	3 ^a^* (9.7)		3 ^a^* (8.3)	3 ^a^* (15.8)		3 ^a^ (12.0)
*Staphylococcus epidermidis*	1 (3.2)		1 (2.8)			
*Staphylococcus hominis*	1 ^a^ (3.2)		1 ^a^ (2.8)			
*Streptococcus constellatus*		1* (20.0)	1* (2.8)			
*Streptococcus viridans*	1 * (3.2)		1* (2.8)			
*Streptococcus pneumoniae*	1 (3.2)		1 (2.8)			
*Group A Streptococcus*		1 (20.0)	1 (2.8)			
*Gram-positive bacilli*				2 (10.5)		2 (8.0)
Gram negative						
*Nesseria gonorrhea*		1 (20.0)	1 (2.8)			
*Aeromonas salmonicida*				1 (5.3)		1 (4.0)
*Propionibacterium acnes*	1 (3.2)		1 (2.8)			
*Haemophilus influenzae*	2 * (6.5)		2 * (5.6)			
*Citrobacter koseri*				1 * (5.3)		1 (4.0)
*Eikenella corrodens*		1 * (20.0)	1 * (2.8)			

^a^ The organisms were isolated from blood culture. * The organisms marked with an asterisk are associated with monomicrobial or polymicrobial infection. Abbreviations: MSSA, methicillin-susceptible *Staphylococcus aureus*; MRSA, methicillin-resistant *Staphylococcus aureus*; CoNS, coagulase-negative staphylococci.

**Table 5:** Efficacy of antibiotics against the commonly isolated pathogens, MSSA, MRSA, and other staphylococcal and streptococcal species.

	2000–2009			2010–2019		
Antibiotics	Sensitive No.	Resistant No.	Sensitivity Rate (%)	Sensitive No.	Resistant No.	Sensitivity Rate (%)
Imipenem	2	0	100	17	0	100
Levofloxacin	20	0	100	17	0	100
Rifampin	21	0	100	16	0	100
Vancomycin	21	0	100	17	0	100
Trimethoprim/ sulfamethoxazole	19	3	86.4	17	0	100
Minocycline	11	5	68.8	6	0	100
Chloramphenicol	7	13	35.0	10	7	58.8
Clindamycin	5	16	23.8	5	12	29.4
Erythromycin	4	18	18.2	5	12	29.4
Oxacillin	10	11	47.6	2	15	11.8
Penicillin	1	18	5.3	0	4	0
**Sensitivity Rate (%) = sensitive no./(sensitive no. + resistant no.) × 100**						

Abbreviations: MSSA, methicillin-susceptible *Staphylococcus aureus*; MRSA, methicillin-resistant *Staphylococcus aureus.*

## Data Availability

All data are available upon a reasonable request from the authors.
